# Overfat and Underfat: New Terms and Definitions Long Overdue

**DOI:** 10.3389/fpubh.2016.00279

**Published:** 2017-01-03

**Authors:** Philip B. Maffetone, Ivan Rivera-Dominguez, Paul B. Laursen

**Affiliations:** ^1^MAFF FITNESS PTY LTD., Sydney, Australia; ^2^Sports Performance Research Institute New Zealand (SPRINZ), AUT University, Auckland, New Zealand

**Keywords:** obesity, body mass index, overweight, cachexia, chronic illness, World Health Organization, waist circumference, mortality

## Abstract

For the first time in human history, the number of obese people worldwide now exceeds those who are underweight. However, it is possible that there is an even more serious problem—an overfat pandemic comprised of people who exhibit metabolic health impairments associated with excess fat mass relative to lean body mass. Many overfat individuals, however, are not necessarily classified clinically as overweight or obese, despite the common use of body mass index as the clinical classifier of obesity and overweight. The well-documented obesity epidemic may merely be the tip of the overfat iceberg. The counterpart to the overfat condition is the *underfat* state, also a common and dangerous health circumstance associated with chronic illness and starvation. Currently (and paradoxically), high rates of obesity and overweight development coexist with undernutrition in developing countries. Studies in cognitive linguistics suggest that accurate, useful, and unintimidating terminology regarding abnormal body fat conditions could help increase a person’s awareness of their situation, helping the process of implementing prevention and simple remedies. Our contention is that promoting the terms “overfat” and “underfat” to describe body composition states to the point where they enter into common usage may help in creating substantive improvements in world health.

## Introduction

Obesity, together with the overweight condition, continues to be a serious global epidemic that significantly affects population health and economy (1). Obesity affects people of all ages and incomes throughout the world. In the last three decades, no country has reduced obesity rates, which are expected to continue increasing as incomes rise in low- and middle-income countries in particular (2). Most of these statistics have been derived using the most frequently used proxy for body composition estimate, the body mass index (BMI).

The estimation of body fat composition has been studied in various populations around the world. The accuracy of these statistics may be partly skewed by the variety of different indirect methods of body composition assessment (3–5). However, most of these studies showed average body fat percentages above healthy recommended levels reported by Bray (6), which, for example, are 21–32% for 21- to 39-year-old normal-weight women and 8–20% for men of the same age and BMI category.

It is well documented that serious limitations exist when estimating body composition using BMI, due to the fact that it underestimates adiposity (body fat percentage) levels in the population at large (7–10). Different ethnic and gender cutoffs for BMI also exist (11). The morbidity and downstream diseases associated with obesity—metabolic syndrome, cardiovascular disease (12, 13), and in fact most non-infectious chronic diseases (14)—are pathophysiological consequences of excess adiposity.

The metabolic underpinnings of excess adiposity (15), as well as its many adverse consequences (16), have been documented to lead to states of chronic illness later in life (12, 13). These states are often characterized by loss of lean muscle mass (sarcopenia) and loss of both lean and fat mass (FM) (cachexia) (17–20). Sarcopenia is another serious global condition (17–20), and cachexia is now considered a public health crisis (20), whose incidence may be underestimated (21). In light of the probable underestimation of overfat individuals, this health crisis becomes compounded: this has created a population of overfat individuals whose health needs may be unmet and overlooked. Combined, this exacerbates future rates of chronically ill, sarcopenic and cachexic individuals—another health crisis in its own right.

The widespread, institutionalized underestimation of adiposity levels, caused by the blanket adoption of BMI as the main indicator of obesity, poses serious challenges to the accurate diagnosis, prevention, and treatment of obesity-related diseases (8). We argue that new methods and associated terms are needed to more appropriately identify a person’s adiposity.

More appropriate terms could help all those in public health and those using health services. Adopting new terminology has been argued on the grounds that more descriptive terminology has a downstream positive effect on the systems and practices that surround the phenomenon (22, 23). It is well established that, in addition to health practitioners, health consumer/patient opinions should be considered in the design, delivery, and evaluation of health services, with consumer expectations as one of the criteria used by the World Health Organization (WHO) to evaluate health system performance (24, 25). As one example Sav et al. (24) use the term *consumer* instead of *patient*, believing that the latter term can be disempowering.

Herein, we define new *overfat* and *underfat* conditions, estimate their worldwide frequencies, and argue for the importance of implementing these terms into popular and scientific literature. Our contention is that promoting the terms *overfat* and *underfat* to the point where they enter into common usage may help in creating substantive improvements in world health.

## Clinical Markers of Body Composition

The present standard for gaining an estimate of body composition in the clinical setting is through measurement of the BMI; an individual’s weight in kilogram divided by their height in square meter. Different standardized cutoff points indicate the clinical diagnoses of being underweight, of normal weight, overweight, or obese, according to the following demarcation points (in kilograms per square meter) (9, 26):

- <18.5 = underweight
- 18.5–24.9 = normal weight
- 25–29.9 = overweight (pre-obese)
- >30 = obese.

While BMI is a reasonable general indicator of body fat (made up mostly of triacylglycerols) and WHO defines overweight and obese as abnormal or excessive fat accumulation for both adults and children (26), it does not measure body fat directly. The US Center for Disease Control and Prevention recommends against using BMI as a diagnostic tool (27) and instead recommends that it can be used as a measure to track weight status in populations and as a screening tool to identify potential weight problems in individuals. Indeed, high BMI levels are associated with future health risks, including the prediction of morbidity and death (27).

According to the most comprehensive BMI trend analysis to date, global obesity numbers have risen dramatically, from 105 million adults in 1975 to 641 million in 2014. Worldwide, the age-corrected proportion of men who were obese climbed from 3.2 to 10.8% in that time and the rate among women more than doubled, from 6.4 to 14.9%. During the same 40-year period, the proportion of men who were underweight globally fell from 13.8 to 8.8% and among women declined from 14.6 to 9.7% (1, 28). Obese and overweight children also make up a rapidly increasing population, with Lobstein et al. (29) estimating ~12% of children worldwide aged 5–17 years being affected with annual increases of 1%. Prevalence of obesity alone in US children aged 2–19 years is estimated at 17% (30).

Importantly, however, these statistics do not always include the population that is overweight. When the incidence of being overweight is included within obesity statistics, rates of excess weight have been shown to be dramatically greater. Indeed, the WHO has reported that, as a group, the obese and overweight adult population is now approaching 40% of the world’s population (26), with rates in the US now at ~66% (31).

## Problems with BMI as a Measure of Obesity

The fundamental problem with using the measurement of one’s height and weight to indirectly ascertain an individual’s body composition is that high percent body fat values are often associated with lower BMI categories (8). In addition, a person’s body weight is in itself unlikely to be a primary contributor to the negative health effects that are associated with obesity. Obesity of course is now accepted as merely a sign of a metabolic phenomenon associated with unhealthy changes in levels of body fat (15, 32).

The limitations associated with using BMI clinically have been well documented. As one example, Tomiyama et al. (9) showed that 30% of individuals classified as being healthy based on their BMI scores were in fact metabolically unhealthy based on clinical evaluations. In another study, nearly 50% of men and women classified as obese using densitometric methods were determined to be of normal BMI (33). Athletes who build high levels of muscle (lean body) mass often possess BMI levels above 30 and would be inappropriately classified as obese (34). However, when they retire, many will increase their body fat levels substantially, while maintaining their high BMI. A study of 926 retired NFL players whose mean BMI was 30 and mean waist circumference was 40 showed that 61% were at risk for atherosclerosis; the most at risk being those with larger waist circumference (10). A number of other studies have reported various discrepancies between BMI and the accumulation of body fat in various other populations (10, 35–37). Clearly, BMI has limited relevance for assessing accurate markers of body composition and body fat distribution, which are important signs of metabolic health (1).

## Is Measurement of Waist Circumference a More Practical Solution for Clinical Identification of Metabolic Health Issues?

The WHO is currently undertaking a review and assessment of available data on the relationship between waist circumference, morbidity, health risk, and the interaction with BMI measurements (38). Waist circumference, more than BMI, has a very strong association with health risk (12) and is part of a series of practical tools that have greater assessment value than BMI, including blood pressure, triglyceride, glucose, insulin resistance, and C-reactive protein (9, 37). These tests could appropriately replace BMI in both a clinical and, in the case of waist circumference, in the home environment to help measure and monitor body fat as a way to improve health and reduce the risk of mortality (37, 39). In view of tests such as waist circumference, it is striking that the most prolific measure used to ascertain a body composition level that is likely to be not healthy is BMI (40), a measurement that does not distinguish between adipose tissue and other tissues of the body.

Waist circumference has increased significantly in both children and adults worldwide over the past 20–25 years (41). Some of these increases are greater than what would be expected based on changes in BMI (42). As another example, a Canadian government health survey evaluated 6,306 healthy people aged 6–79 years between 2009 and 2011 using waist circumference (43) and showed that up to 50% of men, 70% of women, 20% of boys, and 41% of girls had increased or high health risk based on National Institutes of Health measurement guidelines (44).

## The Terminology of Obesity and Its Implications

A contributing condition to this unfortunate reality may be the fact that current terminology used to describe the state of being overfat and its related metabolic conditions centers on *weight*, not *fat*. As Baumgartner et al. (45) write: “There are very few reports of associations between more exact measures of body fatness, such as percent body fat (%BF) or FM, and chronic disease risk.” For this reason, various authors (46–48) have argued that definitions of obesity based on height and weight be modified.

### Conflation of “Weight” and “Fat”

Compounding the problem further is that researchers and health-care practitioners have been known to treat terms relating to weight the same as those that relate to fat. Two examples of this are shown in recent reports appearing in popular online websites. Both sources reported on a new study published in *JAMA Internal Medicine* showing that in monozygous twins, higher BMI was not associated with increased risk of heart attack, but was associated with increased risk of type 2 diabetes (49). The *American Heart Association News* quoted the study’s lead researcher Peter Nordstrom as saying, “The fatter twin actually had a lower risk of myocardial infarction or death, although risk of diabetes was higher” (50). The published study (49) does not address body fat, but uses the words “heavier,” “leaner,” “fatter,” and “obese” interchangeably throughout. Another article about this study from http://ScienceDaily.com stated that “all twins in the study had different levels of body fat” (51), when of course, body fat was not assessed.

### Social Factors Affecting the Use of Terminology

While the perception of healthy body size and composition differs worldwide, within ethnic groups, cultures, and even the health-care community (20), many of the terms that surround obesity (such as “fat” and “obese”) are often viewed as personal insult (52, 53). In a study on perceptions of obesity terminology in childhood, it was found that doctors’ use of terms related to weight (“unhealthy weight,” “weight problem,” and “weight loss”) was seen as far more desirable by parents than those related to “fat” or “obesity,” which were viewed more negatively (54). Interestingly, however, Puhl et al. (54) ranked this terminology with respect to parents’ perceptions of the level that each term would motivate their children to lose weight and found an inverse correlation between perceived stigma and motivation. That is, the most stigmatizing terms were viewed as the least motivating, whereas the least stigmatizing terms were the most motivating (54). However, Tailor and Ogden (55) found that healthy subjects were more likely to be offended by doctors’ use of the term *obese*, whereas obese patients found euphemisms more upsetting. Thus, healthy and obese patients appear to have the opposite response to the same terminology. In fact, Tailor and Ogden (55) speculate that the meaningfulness of the term “obesity,” what healthy subjects might refer to as a “stigma” may itself help drive obese patients’ desire to improve their health status.

The perception that the most stigmatizing terminology may be the least motivating may be just that: a perception. While such studies may be germane to interactions between patient and doctor, there are undoubtedly many social arenas (e.g., the playground) where the terms found most stigmatizing by Puhl et al. (54) are wielded to the disadvantage of overfat individuals. Nonetheless, it is not unreasonable to suggest that the “weight”-based terminology used to describe the overfat phenomenon gained prevalence over “fat”-based terms precisely due to its euphemistic function. Unfortunately, the euphemistic conflation of “fat” and “weight” may have caused the reality of these metabolic issues to be obscured from health-care professionals and the general public, making it more difficult to create lasting positive changes in the public health sector.

## The Metabolic Phenomenon

The body’s adipose tissue plays an important role in endocrine function, as well as various other systems in the body. The hormonal role of adiposity is so influential that some authors suggest adipose tissue be understood as an endocrine organ (56–58). It is precisely the endocrine dysfunction associated with high body fat that can lead to the pathophysiology of obesity, as well as illnesses with an underweight component such as cachexia. The metabolic phenomena that open the door to excessive accumulation of fat (in the case of obesity) or an unhealthy loss of fat (in the case of being underweight) are causes of the negative signs and symptoms associated with each of these two conditions.

High levels of body fat are associated with low-grade chronic inflammation (59). In turn, such inflammation is associated with various downstream diseases, including type 2 diabetes (60, 61), heart disease (16), cancer (62), stroke (63), Alzheimer’s (64), and others (65, 66). These health epidemics are currently having devastating effects on the world economy. In 2014 for example, skyrocketing health-care costs climbed to $3 trillion in the US alone (67). Worldwide, chronic diseases are estimated to be responsible for a $17.3 trillion cumulative economic loss between 2011 and 2030 due to health-care expenditures, reduced productivity, and lost capital (68).

Conversely, weight itself may play only a tangential role in both the pathophysiology and the symptomatology of obesity, as well as conditions associated with being underweight. While excess weight can directly and adversely affect the musculoskeletal system, there is little indication that excess weight, when not produced by a relative or absolute increase in FM, a relative or absolute decrease in lean mass, or a combination of both, is causally related to the metabolic underpinnings of obesity. Studies on the risk for cardiovascular disease in Football Players—a high-BMI population with large percentages of lean mass—caution that reliance on BMI as a sole determinant of disease risk may be inappropriate (10, 69). This and various other populations may be contributing to the percentage of false-positives observed through the use of BMI to ascertain metabolic disease risk (33). In simpler terms, we can say that weight relates to obesity and being underweight, primarily with respect to the fact that more fat typically (but not always) means more weight, and less fat (typically but not always) means less weight. These problems create confusion for doctors and patients alike and may often play a significant role in reducing quality of life and lifespan in all populations (70).

A critical factor associated with the health of underweight individuals is the loss of fatty tissue and its endocrine and metabolic functions (45, 58). In conditions of malnutrition, such as cancer cachexia and anorexia, there may be significant alterations in the production of many proteins, such as leptin, secreted from adipocytes with important metabolic and regulatory implications (45). Due to the importance of fatty tissue on health outcomes of underweight people, we argue that the phenomenon of being underweight may, to a large degree, relate to being underfat, a condition that may coexist with the loss of lean body mass. Indeed, cachexia, a condition characterized by severe body weight, fat, and muscle loss as a result of chronic illness occurs in about 80% of all cancer patients (21) and is frequent in patients with chronic heart failure, chronic obstructive pulmonary disease, chronic kidney disease, cystic fibrosis, liver cirrhosis, Crohn’s disease, rheumatoid arthritis, stroke, neurological degenerative diseases, and other chronic illness (20).

**Figure 1:**
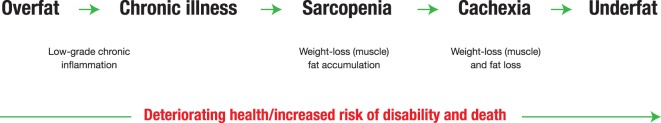
**The overfat–underfat progression**. The common spectrum of deteriorating health during the lifespan from the overfat to underfat state.

Sarcopenia is defined as the progressive loss of type II fast-twitch muscle fibers and strength with aging and is a key public health problem, with the prevalence in the elderly as high as 50% (71). In addition to muscle atrophy, there is an accumulation of fat within existing muscle (72). The combination of higher body fat and sarcopenia has been termed *sarcopenic obesity* and is associated with low-grade chronic inflammation and insulin resistance, among other conditions (73). Sarcopenia increases the risk of disability and poor quality of life and death and may be an important association with chronic illness. Many who become underfat due to chronic illness may in fact owe their underfat status to a previously developed overfat metabolism associated with chronic inflammation and disease, as illustrated in Figure 1. This same metabolic status could lead to cachexia and other forms of unhealthy fat loss later in life (74).

In summary, cachexia and sarcopenia, which are partly defined by loss of muscle mass (72), may occur in part due to unhealthy changes in fatty tissue. The metabolic markers associated with excess fat, such as systemic inflammation, elevated C-reactive protein, and excessive oxidative stress, drive both sarcopenic obesity and cachexia (72, 75). In particular, an imbalance between pro- and anti-inflammatory cytokines may play a key role in the pathogenesis of cachexia (72). Indeed, improvements in fat metabolism through the administration of fatty acids can eliminate in large part the signs and symptoms of sarcopenia and cachexia. For example, eicosapentaenoic fatty acid has been successfully used to stabilize weight in late-stage cancer patients (76) and may function due to its primary effects of reducing inflammation, C-reactive protein, and oxidative stress.

## New Terms

In view of the above, we propose that the terms *overfat* and *underfat* become common for three main reasons:

1. Measuring *adipose* tissue tends to better predict risk for the symptomatology of being *overfat* than measuring weight.
2. The symptomatology of being *overfat* is also found in populations who do not fit the weight-based criteria of obesity, while weight-based measures such as BMI can underestimate the incidence of being overfat and lead to misdiagnosis.
3. Being “underweight” is also importantly a problem. Any of the main health concerns in an underweight population may stem from endocrine dysregulation due to the loss of fatty tissue secondary to chronic illness or starvation, including disordered eating and excess exercise (discussed below).

The term *overfat* accurately specifies the precise problem of excess body fat and impaired fat metabolism that directly influences health and fitness. It also eliminates common terms that are not accurate, including being *overweight* (not a measurement of fat) and *obese*, which references individuals being above a certain BMI (9, 26).

## Who is “Overfat”?

**Figure 2:**
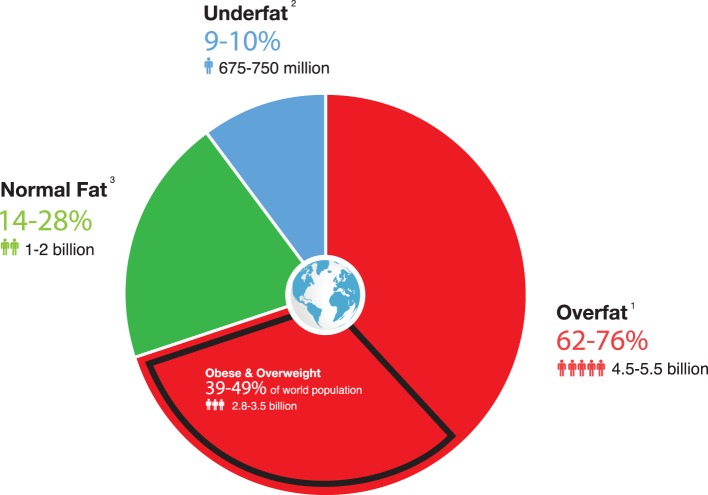
**Estimated number and percentage of overfat and underfat adults and children worldwide (based on 2014 world population numbers of 7.2 billion)**. ^1^Includes obese and overweight and other populations listed above (items 1–5). The 62% number does not include item 6 (children). ^2^Includes 666 million adults due to starvation, plus 10.8 million chronically ill people who were cachexic at time of death in 2008 (with the high range including 70 million with eating disorders). ^3^World population of 7.2 billion minus overfat plus underfat.

While some data are available [reviewed by St-Onge (8)], one problem with estimating the number of overfat people worldwide is the lack of sufficient body fat data associated with the inadequate definitions used to estimate it more accurately. However, extrapolations might be made from other existing data. The notion of a worldwide overfat pandemic that well exceeds that of obesity- and overweight-defined conditions may be based on the facts regarding a variety of different and existing unhealthy states, which include:

1. Adults who are overweight and obese. The WHO has reported that in 2014, 39% of the world’s population was overweight, with 13% being obese (26, 77).
2. Those who are *metabolically obese, normal weight* (MONW) individuals (9, 48, 78) and could include up to 40% of normal-weight individuals. We estimate that the percentage of MONW individuals in the overall population is 20% (2).
3. Individuals with sufficient excess body fat stores to substantially impair health (46) but who are not part of the above categories, and are sometimes labeled as “normal weight obesity” (79). Included are people with deposition of excess fat in organs such as the liver, heart, and muscles that can occur with aging (80).
4. Individuals with sarcopenic obesity (73).
5. Those with increased abdominal fat stores who may not fit the first two categories, as many are not overweight or have high BMIs. Abdominal fat stores are more metabolically active and have a greater impact on health, regardless of the person’s weight (10, 48, 81, 82). Ruderman et al. (48) suggest that many MONW individuals are included in this category. This has been taken into account in our Figure 2 overfat estimate.
6. As of 2014, 17–22% of children. Indeed, in 2004, Lobstein et al. (29) estimated the incidence of being overweight and obese worldwide for children over age 5 at 12%, with an annual increase of 1%. The WHO estimates overweight children under age 5 (2013) to be 6.3%, and that this figure could be 11% by 2025 (77).

If we consider that the percentage of individuals who are either overweight or obese or MONW (categories 1 and 2) is underestimated, the combined number makes up a shockingly large amount of the population (Figure 2). While substantially fewer individuals fit into categories 3, 4, and 5, and we have found no data available to estimate these numbers, such frightening statistics suggest that an unrecognized high percentage of the world population is overfat enough for their health to be negatively impacted.

An important reason for estimating the number of overfat people in the world is that they may represent a common source of chronic disease. This estimation, however, is particularly difficult for a number of reasons. First, appropriate studies that have accurately measured body fat in both adults and children are lacking. Second, using data only for previously categorized obese and overweight individuals may significantly underestimate the number of overfat individuals. Third, data available for obese and overfat children often use different age ranges, making total estimates of overfat children difficult. Finally, adults and children who have not been classified as obese or overweight can still be overfat.

Figure 2 (below) portrays how alarming the overfat pandemic may be when compared to the numbers and percentages of overweight and obese people in the world based on best estimates from the available data (1, 2, 29, 77, 83–87).

## Who is Underfat?

There are two main categories of people at risk for being underfat, in addition to those who have malnutrition due to starvation:

1. Individuals with chronic illness, especially with cachexia: cachexia increases mortality in those with chronic disease and is present in 30% of patients who die, making it one of the major contributors to death worldwide (20). Of the estimated 57 million global deaths in 2008, 36 million (63%) were due to chronic diseases (84).
2. Those with eating disorders: 70 million people worldwide, including those who exercise excessively (anorexia athletica) (85).

While present percentages of starvation-related underfat people are diminishing, it is still a serious disorder in developing nations. During the dramatic rise in obesity over the last four decades, the proportion of underweight adults globally fell from about 14% to about 9% (2). But as the world becomes more westernized, chronically ill people will stay alive longer through medical interventions. In 2010, an estimated 524 million people were aged 65 and older, making up 8% of the world’s population. By 2050, this number may triple to 1.5 billion, making up about 16% of the world population (88). When we include the increasing population of those with eating disorders, including anorexia athletica, we may see future increases in the underfat population, despite reductions of those who are underfat due to starvation. Effectively, this may create a shrinking population of healthy people. The effects of normal aging may compound this situation further. Changes in body composition, resulting from a shift across the overfat conditions toward one of decreased muscle mass in combination with maintained or increased FM (89), may increase rates of sarcopenic obesity, described as the combination of increased body fat in conjunction with reduced muscle mass (66, 72).

**Figure 3:**
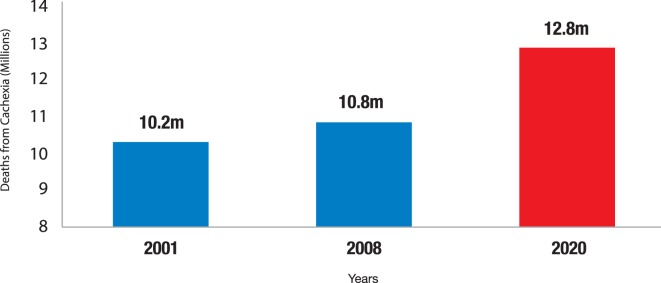
**Increase in death rates from cachexia**. Number of people from cachexia at time of death. If we hypothesize that 60 million deaths from all causes will occur in 2020, WHO extrapolations predict that 21.3% of people (12.8 million) would have cachexia at time of death.

Cachexia is leading to a growing number of underfat people due to increasing rates of chronic disease. Furthermore, the combined increasing global aging population and worldwide westernization further escalates the numbers of underfat people due to non-malnutrition (cachexia and eating disorders). The finding that approximately 30% of those who die from chronic disease have cachexia (20), combined with the statistic that worldwide death rates from chronic disease in 2008 totaled 36 million, suggests that nearly 11 million people who died from chronic disease in 2008 also had cachexia. As mortality from chronic diseases rise (from 60% of all deaths in 2001 to 63% in 2008 to an extrapolated 71% by 2020) (83, 90), in combination with the ballooning elderly population (88), leads us to extrapolate that the figure of 11 million deaths of people with cachexia will increase dramatically (Figure 3).

## Designing Better Terminology

It is not the case that measures of illness (such as BMI) fail or succeed only in terms of how accurately they measure the illness in question. The view that they do overlooks the fact that in addition to being a health phenomenon, public health is a public (that is, a *social*) phenomenon. In this section, we present evidence that whether or not the tropes, ideas, and concepts surrounding a particular public health phenomenon are in fact emblematic of the physiological underpinnings of the actual problem—whether the public mistakes a fat-based phenomenon for a weight-based one—can indirectly drive the success (or failure) of public health initiatives.

The title of the article *Are Normal-weight Americans Over-Fat?* elucidates the extent of the problem (8). In previous sections, we discussed how the relevance of weight to the bulk of metabolic diseases is contingent on fat, and limited to those situations where excess (or a lack of) fat has created excess weight (or a lack thereof). We have found no evidence supporting the notion that when a “normal weight” individual is overfat, consideration of their weight status provides additional information relevant to the determination of their metabolic disease risk. In these cases, the “normal” status of this weight statistic draws attention away from the fact that this person’s relevant metabolic metrics indicate that they are in fact at increased risk of disease. We argue that the notion and mention of an overfat individual’s “normal weight” status provides an unfortunate barrier to the understanding that they are at increased risk of disease, which may dampen the motivation to improve health status, as well as confound the relevance of the strategies used to improve health. Due to the increasing frequency of “normal-weight but overfat” individuals in the world population, we believe that terms relating to, referring to, and including “weight” are effectively functioning as a red herring, obscuring the problem (and its possible solutions).

**Figure 4:**
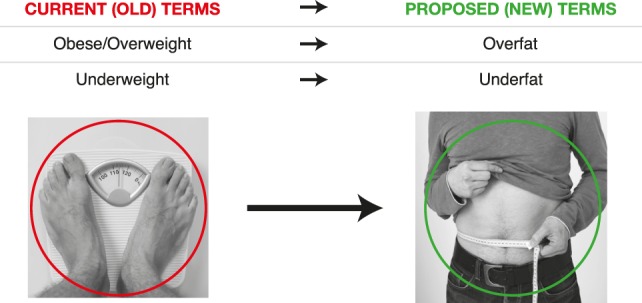
**Changing terminology**.

An online University of Houston document states that, “The terms overfat and underfat are very useful because they describe how much of your total body weight is made up of fat. Underfat means having too little body fat; overfat means having too much body fat. Obesity is a term used to describe people who are very overfat” (91). Introducing the terms *overfat* and *underfat* into the medical lexicon as well as common parlance could have a variety of benefits that include:

1. Describing these serious health issues with terminology that centers and obviates the main issue: unhealthy surpluses and lack of fatty tissue.
2. Clear and transparent terminology helps patients, health practitioners, and the public make better health decisions and set better health goals.
3. Using the term “overfat” may help mitigate the problems associated with those who do not perceive themselves to be at health risk due to having normal BMI (Figure 4).

Studies on semantic priming show that the word-form, or surface-shape, of a particular term (say “overweight”) deeply influences our cognition (92) and drives our attention toward other words, images, and even goals (93), which are semantically related to that word-form (94). While dictionary definitions of being “overweight” often include its cause as excess fat, the shape of the word-form directs attention toward *weight* and away from *fat*. This may make it more likely for individuals to think that weight loss generally, rather than fat loss specifically, will lead to better health.

The view that weight is the primary indicator of health is widespread in the public consciousness. The bathroom scale (which measures weight) is the cultural icon of leanness, fitness, and health. For comparison, the skinfold caliper and other devices (that measure fat) are far less used. Other associations that relate to fat—such as the measurement of waist circumference—are also overshadowed by our familiarity with the bathroom scale. That the prevalence of overweight terminology would co-occur with the cultural dominance of the bathroom scale should come as little surprise. Indeed, the frequency with which we encounter terminology associated with a particular topic or concept increases our ability to recognize it, which in turn drives decision-making (95). Moreover, terminology does not only prime attention. It can also influence decision-making and goal setting without our conscious awareness (93). In fact, it has been suggested that the primary function of attention is to influence our decision-making processes, known as *Goal Contagion* (96).

In light of this, we believe that promoting the term *overfat* to the point where it becomes more common than the term *overweight* (and other health-related terms that refer to weight) will promote greater public understanding that the obesity epidemic is the tip of an overfat iceberg. In turn, the problems associated with being overfat and underfat will be more easily addressed by health-care professionals, public health officials, and the lay public alike.

## Conclusion

Given the many associations between weight, obesity, and poor health in scientific corpora (and the fact that associations with fat are fewer), better terminology is needed to describe the phenomena associated with inappropriate body compositions more accurately, so that people exposed to the term “overfat” versus “overweight” may make improved choices and set better goals *because the term more accurately encapsulates the problem itself*. To improve accuracy, end confusion, and encourage healthier lifestyles that reduce excess body fat, while helping prevent chronic illness, it is proposed that both the scientific and clinical community and the general population, especially the media who encourage the use of specific terms, simplify these two very common states of unhealthiness with simple, straightforward terms, replacing others that are frequently not accurate, sometimes wrong, and often confusing. We propose that promoting the terms “overfat” and “underfat” to the point where they enter into common usage may help in creating substantive improvements in world health.

## Author Contributions

Through his work as a clinician and advocate of health, Dr PM has been developing the ideas described within this manuscript for decades. Through his background expertise in linguistics, IR-D contributed substantially to the terminology issue described in this paper. PL, a professor of sport and exercise physiology, edited and expanded on the concepts the former authors generated.

## Conflict of Interest Statement

PM is the proprietor of MAFF FITNESS PTY LTD, the business that runs a website dedicated to disseminating information pertaining to health, fitness, and longevity (www.philmaffetone.com).

## References

[1] NCD Risk Factor Collaboration. Trends in adult body-mass index in 200 countries from 1975 to 2014: a pooled analysis of 1698 population-based measurement studies with 19.2 million participants. Lancet (2016) 387(10026):1377–96. 10.1016/S0140-6736(16)30054-X 27115820 PMC7615134

[2] Ng M Fleming T Robinson M Thomson B Graetz N Margono C Global, regional, and national prevalence of overweight and obesity in children and adults during 1980–2013: a systematic analysis for the Global Burden of Disease Study 2013. Lancet (2014) 384(9945):766–81. 10.1016/S0140-6736(14)60460-8 24880830 PMC4624264

[3] Li C Ford ES Zhao G Balluz LS Giles WH Estimates of body composition with dual-energy X-ray absorptiometry in adults. Br J Nutr (1999) 82:49–55. 19812179 10.3945/ajcn.2009.28141

[4] Deurenberg P Yap M van Staveren WA. Body mass index and percent body fat: a meta analysis among different ethnic groups. Int J Obes Relat Metab Disord (1998) 22:1164–71. 10.1038/sj.ijo.0800741 9877251

[5] Gallagher D Heymsfield SB Heo M Jebb SA Murgatroyd PR Sakamoto Y. Healthy percentage body fat ranges: an approach for developing guidelines based on body mass index. Am J Clin Nutr (2000) 72(3):694–701. 10966886 10.1093/ajcn/72.3.694

[6] Bray GA Contemporary Diagnosis and Management of Obesity and the Metabolic Syndrome. Newtown, PA: Handbooks in Health Care (2003).

[7] Anker SD von Haehling S. Cachexia as a major underestimated and unmet medical need: facts and numbers. J Cachexia Sarcopenia Muscle (2010) 1(1):1–5. 10.1007/s13539-010-0002-6 21475699 PMC3060651

[8] St-Onge MP. Are normal-weight Americans over-fat? Obesity (Silver Spring) (2010) 18(11):103. 10.1038/oby.2010.103 20978478 PMC3837418

[9] Tomiyama AJ Hunger JM Nguyen-Cuu J Wells C. Misclassification of cardiometabolic health when using body mass index categories in NHANES 2005-2012. Int J Obes (Lond) (2016) 40(5):883–6. 10.1038/ijo.2016.17 26841729

[10] Pokharel Y Basra S Lincoln AE Tucker AM Nambi V Nasir K Association of body mass index and waist circumference with subclinical atherosclerosis in retired NFL players. South Med J (2014) 107(10):633–9. 10.14423/SMJ.0000000000000173 25279867

[11] Hunma S Ramuth H Miles-Chan JL Schutz Y Montani JP Joonas N Body composition-derived BMI cut-offs for overweight and obesity in Indians and Creoles of Mauritius: comparison with Caucasians. Int J Obes (2016) 40(12):1906–14. 10.1038/ijo.2016.176 27698347 PMC5144117

[12] Shen W Punyanitya M Chen J Gallagher D Albu J Pi-Sunyer X Waist circumference correlates with metabolic syndrome indicators better than percentage fat. Obesity (Silver Spring) (2006) 14(4):727–36. 10.1038/oby.2006.83 16741276 PMC1894647

[13] Vatanparast H Chilibeck PD Cornish SM Little JP Paus-Jenssen LS Case AM DXA-derived abdominal fat mass, waist circumference, and blood lipids in postmenopausal women. Obesity (Silver Spring) (2009) 17(8):1635–40. 10.1038/oby.2009.80 19343013

[14] Heymsfield SB Ebbeling CB Zheng J Pietrobelli A Strauss BJ Silva AM Multi-component molecular-level body composition reference methods: evolving concepts and future directions. Obes Rev (2015) 16(4):282–94. 10.1111/obr.12261 25645009 PMC4464774

[15] Tack CJ Stienstra R Joosten LA Netea MG. Inflammation links excess fat to insulin resistance: the role of the interleukin-1 family. Immunol Rev (2012) 249(1):239–52. 10.1111/j.1600-065X.2012.01145.x 22889226

[16] Koenig W Sund M Fröhlich M Fischer HG Löwel H Döring A C-reactive protein, a sensitive marker of inflammation, predicts future risk of coronary heart disease in initially healthy middle-aged men results from the MONICA (Monitoring Trends and Determinants in Cardiovascular Disease) Augsburg Cohort Study, 1984 to 1992. Circulation (1999) 99(2):237–42. 9892589 10.1161/01.cir.99.2.237

[17] Atlantis E Martin SA Haren MT Taylor AW Wittert GA. Inverse associations between muscle mass, strength, and the metabolic syndrome. Metabolism (2009) 58:1013–22. 10.1016/j.metabol.2009.02.027 19394973

[18] Srikanthan P Karlamangla AS. Relative muscle mass is inversely associated with insulin resistance and prediabetes. Findings from the Third National Health and Nutrition Examination Survey. J Clin Endocrinol Metab (2011) 96:2898–903. 10.1210/jc.2011-0435 21778224

[19] Ruiz JR Sui X Lobelo F Morrow JR Jackson AW Sjostrom M Association between muscular strength and mortality in men: prospective cohort study. BMJ (2008) 337:a439. 10.1136/bmj.a439 18595904 PMC2453303

[20] Farkas J von Haehling S Kalantar-Zadeh K Morley JE Anker SD Lainscak M. Cachexia as a major public health problem: frequent, costly, and deadly. J Cachexia Sarcopenia Muscle (2013) 4(3):173–8. 10.1007/s13539-013-0105-y 23539127 PMC3774921

[21] von Haehling S Anker SD Prevalence, incidence and clinical impact of cachexia: facts and numbers – update 2014. J Cachexia Sarcopenia Muscle (2014) 5(4):261–3. 10.1007/s13539-014-0164-8 25384990 PMC4248411

[22] Stewart AE Lord JH. Motor vehicle crash versus accident: a change in terminology is necessary. J Trauma Stress (2002) 15(4):333–5. 10.1023/A:1016260130224 12224806

[23] Faulkner JA. Terminology for contractions of muscles during shortening, while isometric, and during lengthening. J Appl Physiol (2003) 95(2):455–9. 10.1152/japplphysiol.00280.2003 12851415

[24] Sav A McMillan SS Kelly F King MA Whitty JA Kendall E The ideal healthcare: priorities of people with chronic conditions and their carers. BMC Health Serv Res (2015) 15(1):1. 10.1186/s12913-015-1215-3 26666351 PMC4678633

[25] World Health Organization. The World Health Report 2000 – Health Systems: Improving Performance. Geneva: World Health Organization (2000).

[26] WHO. Obesity and Overweight Fact Sheet [Internet]. (2016). Available from: http://www.who.int/mediacentre/factsheets/fs311/en/

[27] CDC. Body Mass Index: Considerations for Practitioners [Internet]. Centers for Disease Control and Prevention (2016). Available from: https://www.cdc.gov/obesity/downloads/BMIforPactitioners.pdf

[28] Frellick M Obesity Now More Common Than Underweight Worldwide. Medscape Medical News. (2016).

[29] Lobstein T Baur L Uauy R Obesity in children and young people: a crisis in public health. Obes Rev (2004) 5(1):4–85. 10.1111/j.1467-789X.2004.00133.x 15096099

[30] Kim J Peterson KE Scanlon KS Fitzmaurice GM Must A Oken E Trends in overweight from 1980 through 2001 among preschool-aged children enrolled in a health maintenance organization. Obesity (2006) 14(7):1107–12. 10.1038/oby.2006.126 16899790

[31] Ogden CL Carroll MD Curtin LR McDowell MA Tabak CJ Flegal KM. Prevalence of overweight and obesity in the United States, 1999-2004. JAMA (2006) 295:1549–55. 10.1001/jama.295.13.1549 16595758

[32] Gaida JE Alfredson H Kiss ZS Bass SL Cook JL. Asymptomatic Achilles tendon pathology is associated with a central fat distribution in men and a peripheral fat distribution in women: a cross sectional study of 298 individuals. BMC Musculoskelet Disord (2010) 11:41. 10.1186/1471-2474-11-41 20196870 PMC2841085

[33] Smalley KJ Knerr AN Kendrick ZV Colliver JA Owen OE. Reassessment of body mass indices. Am J Clin Nutr (1990) 52(3):405–8. 2393001 10.1093/ajcn/52.3.405

[34] Hind K Gannon L Brightmore A Beck B. Insights into relationships between body mass, composition and bone: findings in elite rugby players. J Clin Densitom (2015) 18(2):172–8. 10.1016/j.jocd.2014.11.002 25659180

[35] Lee J Lee JY Lee JH Jung SM Suh YS Koh JH Visceral fat obesity is highly associated with primary gout in a metabolically obese but normal weighted population: a case control study. Arthritis Res Ther (2015) 17(1):79. 10.1186/s13075-015-0593-6 25889813 PMC4381370

[36] Misra A Anoop S Gulati S Body fat patterning, hepatic fat and pancreatic volume of non-obese Asian Indians with type 2 diabetes in North India: a case-control study. PLoS One (2015) 10(10):e0140477 10.1371/journal.pone.0142749 26474415 PMC4608569

[37] Riedl A Vogt S Holle R de las Heras Gala T Laxy M Peters A Comparison of different measures of obesity in their association with health-related quality of life in older adults – results from the KORA-Age study. Public Health Nutr (2016) 1:1–11. 10.1017/S1368980016001270 27337156 PMC10271135

[38] World Health Organization. Waist Circumference and Waist-Hip Ratio: Report of a WHO Expert Consultation. Geneva: World Health Organization (2008).

[39] Tanamas SK Ng WL Backholer K Hodge A Zimmet PZ Peeters A. Quantifying the proportion of deaths due to body mass index- and waist circumference-defined obesity. Obesity (2016) 24(3):735–42. 10.1002/oby.21386 26833753

[40] Dagan SS Segev S Novikov I Dankner R. Waist circumference vs body mass index in association with cardiorespiratory fitness in healthy men and women: a cross sectional analysis of 403 subjects. Nutr J (2013) 12(1):1. 10.1186/1475-2891-12-12 23317009 PMC3564926

[41] Freedman DS Kit BK Ford ES. Are the recent secular increases in waist circumference among children and adolescents independent of changes in BMI? PLoS One (2015) 10(10):e0141056. 10.1371/journal.pone.0141056 26506450 PMC4624430

[42] Freedman DS Ford ES. Are the recent secular increases in the waist circumference of adults independent of changes in BMI? Am J Clin Nutr (2015) 101(3):425–31. 10.3945/ajcn.114.094672 25733625 PMC4609894

[43] Patry-Parisien J Shields M Bryan S Comparison of Waist Circumference Using the World Health Organization and National Institutes of Health Protocols. Statistics Canada (2015). Available from: http://www.statcan.gc.ca/pub/82-003-x/2012003/article/11707-eng.htm 23061265

[44] National Institutes of Health. The Practical Guide to the identification, Evaluation, and Treatment of Overweight and Obesity in Adults. Bethesda, MD: National Institutes of Health (2000).

[45] Baumgartner RN Heymsfield SB Roche AF. Human body composition and the epidemiology of chronic disease. Obes Res (1995) 3(1):73–95. 10.1002/j.1550-8528.1995.tb00124.x 7712363

[46] Ruderman NB Berchtold P Schneider SH Obesity associated disorders in normal weight individuals: some speculations. Int J Obes (1982) 6(1):151–7. 6749721

[47] Ruderman NB Schneider SH Berchtold P The “metabolically-obese,” normal-weight individual. Am J Clin Nutr (1981) 34(8):1617–21. 7270486 10.1093/ajcn/34.8.1617

[48] Ruderman N Chisholm D Pi-Sunyer X Schneider S. The metabolically obese, normal-weight individual revisited. Diabetes (1998) 47(5):699–713. 10.2337/diabetes.47.5.699 9588440

[49] Nordström P Pedersen NL Gustafson Y Michaëlsson K Nordström A. Risks of myocardial infarction, death, and diabetes in identical twin pairs with different body mass indexes. JAMA Intern Med (2016) 176(10):1522–9. 10.1001/jamainternmed.2016.4104 27479111

[50] American Heart Association News. Higher BMI Increases Diabetes Risk But Not Heart Attacks, Twin Study Finds. American Heart Association News. (2016).

[51] Science Daily/Umea University. Higher BMI Not Associated with Increased Risk of Heart Attack or Early Death, Twin Study Shows. ScienceDaily. (2015).

[52] Ogden J Branson R Bryett A Campbell A Febles A Ferguson I What’s in a name? Patient views of the impact and function of a diagnosis. Fam Pract (2003) 20:248–53. 10.1093/fampra/cmg304 12738692

[53] Tayler M Ogden J Doctors use of euphemisms and their impact on patients beliefs about their illness. Patient Educ Couns (2005) 57:321–6. 10.1016/j.pec.2004.09.001 15893215

[54] Puhl RM Peterson JL Luedicke J. Parental perceptions of weight terminology that providers use with youth. Pediatrics (2011) 128(4):e786–93. 10.1542/peds.2010-3841 21949145

[55] Tailor A Ogden J Avoiding the term ‘obesity’: an experimental study of the impact of doctors’ language on patients’ beliefs. Patient Educ Couns (2009) 76:260–74. 10.1016/j.pec.2008.12.016 19167856

[56] Hutley L Prins JB. Fat as an endocrine organ: relationship to the metabolic syndrome. Am J Med Sci (2005) 330(6):2548–56. 10.1097/00000441-200512000-00005 16355012

[57] Kershaw EE Flier JS Adipose tissue as an endocrine organ. J Clin Endocrinol Metabol (2004) 89(6):2548–56. 10.1210/jc.2004-0395 15181022

[58] Trayhurn P Beattie JH. Physiological role of adipose tissue: white adipose tissue as an endocrine and secretory organ. Proc Nutr Soc (2001) 60(03):329–39. 10.1079/PNS200194 11681807

[59] Festa A D’Agostino R Jr Williams K Karter AJ Mayer-Davis EJ Tracy RP The relation of body fat mass and distribution to markers of chronic inflammation. Int J Obes Relat Metab Disord (2001) 25(10):1407–15. 10.1038/sj.ijo.0801792 11673759

[60] Pradhan AD Manson JE Rifai N Buring JE Ridker PM C-reactive protein, interleukin 6, and risk of developing type 2 diabetes. JAMA (2001) 286(3):327–34. 10.1001/jama.286.3.327 11466099

[61] Donath MY Shoelson SE. Type 2 diabetes as an inflammatory disease. Nat Rev Immunol (2011) 11(2):98–107. 10.1038/nri2925 21233852

[62] Coussens LM Werb Z Inflammation and cancer. Nature (2002) 420(6917):860–7. 10.1038/nature01322 12490959 PMC2803035

[63] Lindsberg PJ Grau AJ. Inflammation and infections as risk factors for ischemic stroke. Stroke (2003) 34(10):2518–32. 10.1161/01.STR.0000089015.51603.CC 14500942

[64] Sokolova A Hill MD Rahimi F Warden LA Halliday GM Shepherd CE Monocyte chemoattractant protein-1 plays a dominant role in the chronic inflammation observed in Alzheimer’s disease. Brain Pathol (2009) 19(3):392–8. 10.1111/j.1750-3639.2008.00188.x 18637012 PMC8094842

[65] Cavicchia PP Steck SE Hurley TG Hussey JR Ma Y Ockene IS A new dietary inflammatory index predicts interval changes in serum high-sensitivity C-reactive protein. J Nutr (2009) 139(12):2365–72. 10.3945/jn.109.114025 19864399 PMC2777480

[66] Sakuma K Yamaguchi A. Sarcopenic obesity and endocrinal adaptation with age. Int J Endocrinol (2013) 2013:204164. 10.1155/2013/204164 23690769 PMC3639625

[67] Martin AB Hartman M Benson J Catlin A National Health Expenditure Accounts Team. National health spending in 2014: faster growth driven by coverage expansion and prescription drug spending. Health Aff (2016) 35(1):150–60. 10.1377/hlthaff.2015.1194 26631494

[68] Bloom DE Cafiero E Jané-Llopis E Abrahams-Gessel S Bloom LR Fathima S The Global Economic Burden of Non-Communicable Diseases. Geneva: Program on the Global Demography of Aging (2012).

[69] Tucker AM Vogel RA Lincoln AE Dunn RE Ahrensfield DC Allen TW Prevalence of cardiovascular disease risk factors among National Football League players. JAMA (2009) 301(20):2111–9. 10.1001/jama.2009.716 19470988

[70] Prospective Studies Collaboration Whitlock G Lewington S Sherliker P Clarke R Emberson J Body-mass index and cause-specific mortality in 900 000 adults: collaborative analyses of 57 prospective studies. Lancet (2009) 373(9669):1083–96. 10.1016/S0140-6736(09)60318-4 19299006 PMC2662372

[71] Ali S Garcia JM Sarcopenia, cachexia, and aging: diagnosis, mechanisms, and therapeutic options. Gerontology (2014) 60(4):294–305. 10.1159/000356760 24731978 PMC4112511

[72] Muscaritoli M Anker SD Argiles J Aversa Z Bauer JM Biolo G Consensus definition of sarcopenia, cachexia and pre-cachexia: joint document elaborated by Special Interest Groups (SIG) “cachexia-anorexia in chronic wasting diseases” and “nutrition in geriatrics”. Clin Nutr (2010) 29(2):154–9. 10.1016/j.clnu.2009.12.004 20060626

[73] Stenholm S Harris T Ferrucci L Sarcopenic obesity: definition, etiology, and consequences. Curr Opin Clin Nutr Metab Care (2008) 11(6):693–700. 10.1097/MCO.0b013e328312c37d 18827572 PMC2633408

[74] Morley JE Thomas DR Wilson MM Cachexia: pathophysiology and clinical relevance. Am J Clin Nutr (2006) 83(4):735–43. 16600922 10.1093/ajcn/83.4.735

[75] Mantovani G Madeddu C Macciò A Gramignano G Lusso MR Massa E Cancer-related anorexia/cachexia syndrome and oxidative stress: an innovative approach beyond current treatment. Cancer Epidemiol Biomarkers Prev (2004) 13(10):1651–9. 15466983

[76] Bruera E Strasser F Palmer JL Willey J Calder K Amyotte G Effect of fish oil on appetite and other symptoms in patients with advanced cancer and anorexia/cachexia: a double-blind, placebo-controlled study. J Clin Oncol (2003) 21(1):129–34. 10.1200/JCO.2003.01.101 12506181

[77] World Health Organization. Global Status Report on Noncommunicable Diseases 2014. Geneva: World Health Organization (2014). Available from: http://apps.who.int/iris/bitstream/10665/148114/1/9789241564854_eng.pdf

[78] Weiss R Bremer AA Lustig RH. What is metabolic syndrome, and why are children getting it? Ann N Y Acad Sci (2013) 1281:123–40. 10.1111/nyas.12030 23356701 PMC3715098

[79] Oliveros E Somers VK Sochor O Goel K Lopez-Jimenez F. The concept of normal weight obesity. Prog Cardiovasc Dis (2014) 56(4):426–33. 10.1016/j.pcad.2013.10.003 24438734

[80] Unger RH. Longevity, lipotoxicity and leptin: the adipocyte defense against feasting and famine. Biochimie (2005) 87(1):57–64. 10.1016/j.biochi.2004.11.014 15733738

[81] Koh-Banerjee P Wang Y Hu FB Spiegelman D Willett WC Rimm EB. Changes in body weight and body fat distribution as risk factors for clinical diabetes in US men. Am J Epidemiol (2004) 159(12):1150–9. 10.1093/aje/kwh167 15191932

[82] Suganami T Ogawa Y Role of chronic inflammation in adipose tissue in the pathophysiology of obesity. Nihon Rinsho (2013) 71(2):225–30. 23631197

[83] World Health Organization. The World Health Report 2002: Reducing Risks, Promoting Healthy Life. Geneva: World Health Organization (2002). Available from: http://www.who.int/whr/2002/en/

[84] World Health Organization. Global Status Report on Non-Communicable Diseases 2010. Geneva: World Health Organization (2011).

[85] Renfrew Center Foundation for Eating Disorders. Eating Disorders 101 Guide: A Summary of Issues, Statistics and Resources. Renfrew Center Foundation for Eating Disorders (2002, 2003). Available from: http://www.renfrew.org

[86] World Health Organization. Global Health Observatory (GHO) Data. (2016). Available from: http://www.who.int/gho/ncd/risk_factors/overweight/en/

[87] Jiang S Lu W Zong X Ruan H Liu Y Obesity and hypertension (review). Exp Ther Med (2016) 12(4):2395–9. 10.3892/etm.2016.3667 27703502 PMC5038894

[88] National Institute on Aging, National Institutes of Health. Why Population Aging Matters: A Global Perspective. Bethesda: U.S. National Institute on Aging (2007).

[89] Melton LJ III Khosla S Crowson CS O’Connor MK O’Fallon WM Riggs BL Epidemiology of sarcopenia. Am Geriatr Soc (2000) 48(6):625–30. 10.1111/j.1532-5415.2000.tb04719.x 10855597

[90] World Health Organization. The World Health Report 1998. Life in the 21st Century: A Vision for All. Geneva: World Health Organization (1998).

[91] University of Houston. Body Composition [Internet]. (2007) Available from: http://www.uh.edu/fitness/PPTs/bodycomp.pdf

[92] Dehaene S Naccache L Le Clec’H G Koechlin E Mueller M Dehaene-Lambertz G Imaging unconscious semantic priming. Nature (1998) 395(6072):597–600. 10.1038/26967 9783584

[93] Bargh JA Gollwitzer PM Lee-Chai A Barndollar K Trötschel R The automated will: nonconscious activation and pursuit of behavioral goals. J Pers Soc Psychol (2004) 81(6):1014 10.1037/0022-3514.81.6.1014 PMC3005626 11761304

[94] Thompson-Schill SL Kurtz KJ Gabrieli JD Effects of semantic and associative relatedness on automatic priming. J Mem Lang (1998) 38(4):440–58. 10.1006/jmla.1997.2559

[95] Klein GA A Recognition-Primed Decision (RPD) Model of Rapid Decision Making. New York: Ablex Publishing Corporation (1993).

[96] Aarts H Gollwitzer PM Hassin RR. Goal contagion: perceiving is for pursuing. J Pers Soc Psychol (2004) 87(1):23. 10.1037/0022-3514.87.1.23 15250790

